# A Novel Choice to Correct Inflammation-Induced Anemia in CKD: Oral Hypoxia-Inducible Factor Prolyl Hydroxylase Inhibitor Roxadustat

**DOI:** 10.3389/fmed.2020.00393

**Published:** 2020-08-06

**Authors:** Zhipeng Yan, Gaosi Xu

**Affiliations:** Department of Nephrology, The Second Affiliated Hospital of Nanchang University, Nanchang, China

**Keywords:** chronic kidney disease, roxadustat, inflammation, hypoxia inducible factor, erythropoietin

## Abstract

Anemia is a complication of chronic kidney disease (CKD), primarily due to insufficient secretion of erythropoietin (EPO) by the kidney. Erythropoiesis-stimulating agents (ESAs) are used to treat anemia associated with chronic kidney disease. A poor response to ESAs has been associated with inflammation. Inflammation can affect erythrocytes and its production in many ways, but mainly through the inflammatory cytokine IL-6 to stimulate the synthesis of hepcidin in the liver. Hepcidin causes iron insufficiency, which causes erythrocytes to fail to mature normally. In addition, inhibition of bone marrow erythroid precursor cells by inflammatory cytokines such as IL-1 and TNF-α also affects bone marrow hematopoiesis. These cytokines are also important factors leading to EPO resistance. Roxadustat is a new drug for the treatment of renal anemia. In addition to promoting the production of EPO, clinical trials have shown that it can significantly reduce hepcidin and can potentially be used for the treatment of inflammation-induced anemia in CKD.

## Introduction

Anemia is one of the major complications of chronic kidney disease (1, 2), which seriously influences the prognosis of patients. The more severe the anemia, the higher the risk of mortality, cardiovascular events and hospitalization (3, 4). The main cause of renal anemia is renal damage, which affects the secretion of erythropoietin. Therefore, when ESAs appeared, the treatment of renal anemia has been improved, with improved quality of life of patients and improved patient survival.

Approximately 5 to 10% of patients with renal anemia have EPO resistance (5), which leads to the need for higher doses of EPO to achieve hemoglobin levels, with cardiovascular events, mortality and other adverse event gradually increasing (6–8). In earlier studies, plasma C-reactive protein (CRP) was usually higher in patients with ESA resistance (9, 10), and many inflammatory cytokines such as IL-6 and tumors necrosis factor-α (TNF-α) were elevated in plasma and highly related to with ESA resistance (11–14). Inflammation may be an important reasons of ESA resistance (15, 16).

Inflammation is another cause of renal anemia (1, 2). Cross-sectional data from 7,389 adult outpatients suggested that an increase in plasma CRP concentration was associated with a decrease in hemoglobin (Hb), and as CKD progresses, inflammation may gradually increase (17). Inflammation has been found in ~35 to 65% of hemodialysis patients (18). Costa et al. found serum CRP and neutrophils were significantly elevated in hemodialysis patients with CKD (19). Inflammatory anemia is a common feature of patients in patients with advanced chronic kidney disease and an established risk factor for end-stage renal disease (20). In patients with inflammatory anemia, ESA do not achieve satisfactory results, and an alternative therapy is needed, especially in patients with advanced chronic kidney disease.

Roxadustat is a new oral anti-renal anemia medication developed by FibroGen. It was approved for marketing in China in December 2018 and China became the first country in the world to use it for the treatment of renal anemia (21). It promotes the production of endogenous erythropoietin, is effective in the treatment of renal anemia, is well-tolerated and safe. This article reviews the related research of Roxadustat, focusing on roxadustat for the treatment of inflammatory anemia of chronic kidney disease.

## Physiological Hematopoiesis and Iron Metabolism

Under normal circumstances, EPO in plasma promotes the differentiation of bone marrow hematopoietic stem cells into erythroid progenitor cells, which then gradually form mature new red blood cells, thereby continuously renewing the body's blood. In this process, plasma transferrin and serum iron are transported to the surface of the red blood cell membrane to bind the transferrin receptor, and enter the cell to release iron through the effect of puffing. Intracellular iron combines with iron porphyrins in mitochondria to form hemoglobin, and complete the maturation of red blood cells.

During the process of maintaining serum iron homeostasis, reticuloendothelial cells swallow senescent red blood cells to reabsorb iron in hemoglobin, and transport intracellular iron to the serum to supplement serum iron with ferroportin (FPN) (22). In addition to the human duodenum, divalent metal transporter 1 (DMT1) and duodenal cytochrome b (DcytB) are important components of the intestinal absorption of iron, especially when body are deficient in iron, which coordinate with each other to absorb the iron in food, and then transports it into the duodenal cells. The iron in the intestinal cells outputs the iron to the blood circulation through FPN to maintain the steady state of the iron in the body. Therefore, increased iron absorption in the intestine is closely related to DMT1, DcytB, and FPN protein levels (23).

## The Effect of Inflammation on the Process of Erythrocyte Production

Like many chronic diseases, chronic kidney disease is largely considered an inflammatory disease (24). Inflammation can affect hematopoietic function. Inflammation promotes hepatic secretion of hepcidin through the interleukin 6 (IL-6) -STAT3 pathway (25–29) and the bone morphogenetic protein (BMP) -SMAD pathway (30). Hepcidin increases binding to iron transporters on monocyte phagocytic and duodenal cell membranes, thereby promoting the internalization and degradation of FPN (31), resulting in obstructed iron output in monocyte macrophages and duodenal cells (32). At the same time, it was found that hepcidin can also inhibit the secretion of DMT1 and DcytB to affect the absorption of intestinal iron (33–36). These factors lead to insufficient plasma iron, which cannot meet the iron required for erythropoiesis to lead to anemia, so hepcidin is the key to causing anemia and ESA resistance (37). A cross-sectional study of serum Hepcidin-25 levels and anemia in non-diabetic chronic kidney disease patients in Japan showed that serum hepcidin was negatively correlated with hemoglobin and gradually leaded to the development of chronic kidney disease and gradually increases (38).

**Figure 1:**
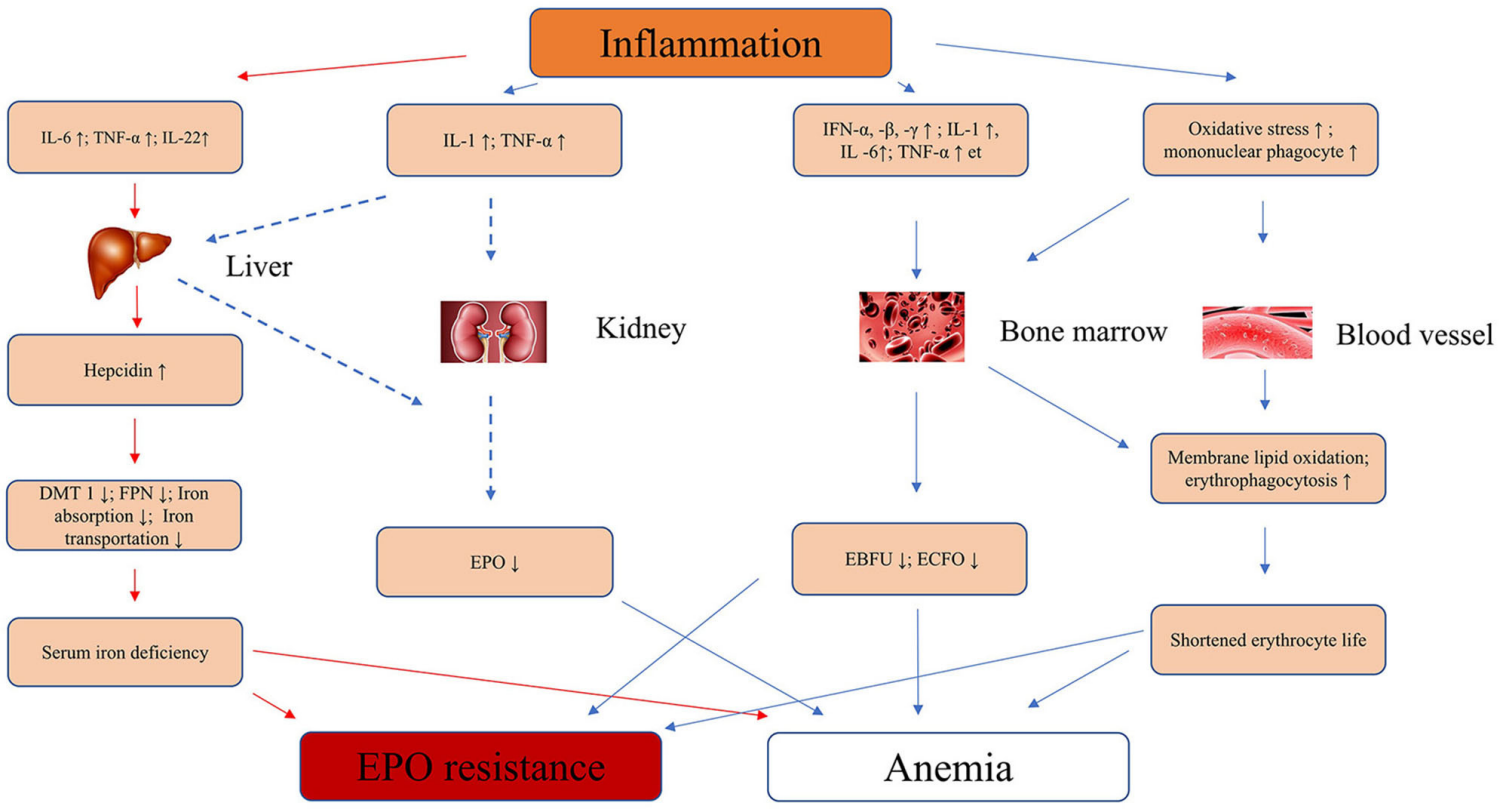
Inflammation leads to anemia and EPO resistance of mechanisms in CKD. IL-6, interleukin-6; IL-1, interleukin-1; IFN-α, -β, -γ, interferon-α, -β, -γ; TNF-α, tumor necrosis factor-α; EPO, erythropoietin; EBFU, erythroid burst-forming units; ECFO, erythroid colony-forming units; ↑, strengthen or increase; ↓, decrease or inhibit; FNP, ferroportin; DMT1, divalent metal transporter 1; CKD, chronic kidney disease.

Second, inflammatory cytokines play an important role in many patients with renal anemia. Inflammatory cytokines IL-1, TNF-a, and IFN-α inhibited the proliferation and differentiation of erythroid precursors in the bone marrow, especially erythroid burst forming units and erythroid colony forming units. In early *in vitro* experiments, IL-1 and TNF-α were found to inhibit erythropoietin expression in isolated rat kidney and human liver cancer cells (39–41), but it has not been confirmed in humans whether inflammatory factors inhibit EPO production. Increased oxidative stress under inflammation leads to lipid peroxidation of erythrocyte membrane (42, 43), at the same time, inflammatory cytokines promote phagocytosis of macrophages (44), which together lead to shortened red blood cell lifespan. Khalil et al. used anti-oxidant vitamin C in hemodialysis patients with inflammatory anemia, and the results showed that they can reduce the EPO resistance of hemodialysis patients and reduce the dose required for EPO (45). Therefore, inflammation can lead to anemia in patients with chronic kidney disease through many ways. Figure 1 summarized the mechanisms by which inflammation causes anemia and EPO resistance.

## Roxadustat of Mechanism in the Human Body

Roxadustat (FG-4592) is a hypoxia-inducible factor prolyl hydroxylase inhibitor (PHI). The main mechanism of action is to inhibit the activity of prolyl hydroxylase domain protein (PHD) under the normoxia. Hypoxia-inducible factor α (HIF-α) has three subtypes 1, 2, and 3, and HIF-β has only one subtypes. In the case where PHD is inhibited, the HIF-α will be not hydroxylated by PHD, nor will it be ubiquitinated by the von Hippel-Lindau tumor suppressor (VHL), It will not be degraded by the proteasome. Therefore, more stable HIF-α enters the nucleus and forms heterodimers with HIF-β (46, 47), which activates target gene expression, such as erythropoietin, glycolysis, and angiogenic factors (48, 49). Therefore, besides being used to treat anemia, roxadustat may also be used to treat other diseases.

## Roxadustat: a New Option for Treating Inflammatory Anemia in CKD

Until now, more than 9,000 patients with renal anemia have participated in phase 2 and phase 3 clinical studies, including non-dialysis patients, hemodialysis patients, and peritoneal dialysis patients. Clinical trials have shown that roxadustat corrected anemia by stimulating the expression of endogenous erythropoietin, which promoted erythropoiesisb and was then non-inferior and superior to placebo and epoetin. No serious drug-related adverse events had occurred in clinical trials to date. Tables 1, 2 were the summary of roxadustat in clinical trials, respectively. Table 3 showed the effects of inflammatory on roxadustat in clinical trials.

**Table 1:** Roxadustat in phase II clinical trial.

**Study**	**Year**	**Nation**	**Study cohort**	**Treatment duration**	**Control group**	**Roxadustat dose (mg)**	**Outcome**	**Hb of roxadustat vs. placebo/epoetin alfa**	**TEAT of FG-4592 vs. placebo/epoetin alfa**
NCT01964196 (50)	2017	China	87 DD-CKD patients	6 weeks	Epoetin alfa	1.1–1.8 mg/kg (low) 1.5–2.3 mg/kg (medium) 1.7–2.3 mg/kg (high)	Maintain Hb of rate	59.1, 88.9% (*P* = 0.008) and 100% (*P* = 0.0003) of the low-, medium-, high dose groups and 50% of the epoetin alfa.	FG-4592 vs. epoetin alfa, 43 and 18%, respectively
NCT01599507 (50)	2017	China	91 NDD-CKD patients	8 weeks	Placebo	1.1–1.75 mg/kg (low) 1.5–2.25 mg/kg (high)	Hb increase ≥ 1 g/dL	Occurred in 23.3% in the placebo group and 80.0%, 87.1% in the low-, high-dose groups.	FG-4592 vs. placebo, 59 and 63%, respectively
NCT00761657 (51)	2019	Japan	107 NDD-CKD patients	24 weeks	Placebo	50, 70, 100 mg	Rate of rise Hb (g/dL/week), maintain Hb of rate	−0.052 (g/dL/week) for placebo and 0.200, 0.453, and 0.570 (g/dL/week) for roxadustat 50, 70, 100 mg groups, respectively; 14.8% for placebo and 81.5, 100, and 100% for roxadustat 50, 70, 100 mg groups	FG-4592 vs. placebo, 88.5 and 70.4%, respectively
NCT00761657 (52)	2015	The USA	116 NDD-CKD patients	16 weeks	Placebo	0.7, 1.0, 1.5, 2.0 mg/kg	Rate of Hb response (ΔHb ≥ 1.0 g/dL)	Hb response and Hb levels increased in a dose-related manner for FG-4592 and both were superior to placebo	FG-4592 vs. placebo, 59 and 46%, respectively

*This table summarizes the efficacy and safety of roxadustat in the treatment of anemia of CKD in Phase II clinical trials. NDD-CKD, non-dialysis chronic kidney disease; DD-CKD, dialysis chronic kidney disease; Hb, hemoglobin; FG-4592, roxadustat; Maintain Hb of rate: Hb level maintain at 10 to 12 g/dL; maintain Hb of rate: Hb level maintain at no 0.5 g/dL below mean baseline value*.

**Table 2:** Roxadustat in phase III clinical trials.

**Study**	**Nation**	**Completion year**	**Number**	**Control group**	**Treatment duration**	**Outcomes**
NCT02652806 (53)	China	2017	305 DD-CKD	Epoetin alfa	26 weeks	(1) ΔHb: roxadustat vs. epoetin alfa, 0.8 [1.1] g/dl vs. 0.5 [1.0] g/dl; *p* = 0.04; (2) Roxadustat was non-inferior and superiority to epoetin alfa
NCT02952092	Japan	2018	303 DD-CKD	Darbepoetin alfa	24 weeks	(1) Hb level of rate (10–12g/dL): roxadustat vs. darbepoetin alfa; 95.2, 91.3%, respectively; (2) Mean Hb level of roxadustat: 10.99 g/dL; (3) Roxadustat was non-inferior to darbepoetin alfa
NCT02174731	The USA et	2018	2133 DD-CKD	Epoetin alfa	52 weeks	(1) Roxadustat reached the primary efficacy endpoint; (2) Roxadustat was superiority to epoetin alfa to increase Hb
NCT02273726	The USA	2018	741 DD-CKD	Epoetin alfa	3.5 years	(1) ΔHb: roxadustat vs. epoetin alfa, 0.39 vs. −0.09g/dl (95% CI, 0.37 to 0.59; *P* < 0.0001); (2) Roxadustat was non-inferior and superiority to epoetin alfa
NCT02052310	The USA et	2018	1043 DD-CKD	Epoetin alfa	4.4 years	(1) ΔHb: roxadustat vs. epoetin alfa, 2.57 vs. 2.36 g/dl (95% CI, 0.08 to 0.29; *P* < 0.0005); (2) Response rate: roxadustat vs. epoetin alfa, 88.2% vs. 84.5% (95% CI, −0.9 to 7.6%); (3) Roxadustat was non-inferior and superiority to epoetin alfa
NCT02174731	The USA et	2018	2133 DD-CKD	Epoetin alfa	52 weeks	(1) Roxadustat reached the primary efficacy endpoint; (2) Roxadustat was superiority to epoetin alfa to increase Hb
NCT02273726	The USA	2018	741 DD-CKD	Epoetin alfa	3.5 years	(1) ΔHb: roxadustat vs. epoetin alfa, 0.39 vs. −0.09 g/dl (95% CI, 0.37 to 0.59; *P* < 0.0001); (2) Roxadustat was non-inferior and superiority to epoetin alfa
NCT02652819 (54)	China	2017	154 NDD-CKD	Placebo	8 weeks	(1) ΔHb: roxadustat vs. placebo, 1.9 [1.2] g/dl vs. −0.4 (0.8) g/dl, *P* < 0.0001; (2) Roxadustat was non-inferior and superiority to placebo
NCT02174627	Europe, the USA et	2018	2781 NDD-CKD	Placebo	52 weeks	(1) Roxadustat reached the primary efficacy endpoint
NCT01750190	The USA	2018	922 NDD-CKD	Placebo	4.5 years	(1) ΔHb: Roxadustat vs. placebo, 2.00 vs. 0.16 g/dl, *P* < 0.0001; (2) Hb level of rate (10–12 g/dL): roxadustat vs. placebo, 86% vs. 6.6%
NCT02780726	Japan	2017	56 PD	NA	24 weeks	(1) Roxadustat maintained the Hb levels within the target range in CKD patients with anemia
NCT02278341	Europe	2018	838 ESRD	Epoetin alfa, Darbepoetin alfa	52 weeks	NA
NCT02780141	Japan	2017	75 HD-CKD	NA	24 weeks	NA
NCT02964936	Japan	2018	100 NDD-CKD	NA	24 weeks	NA
NCT02779764	Japan	2017	167 HD	NA	24 weeks	NA
NCT02988773	Japan	NO completion	344 NDD-CKD	Darbepoetin alfa	52 weeks	NA

*This table summarizes the III trials and main results of roxadustat in the treatment of renal anemia. DD-CKD: undergoing dialysis chronic kidney disease; NDD-CKD, non-dialysis chronic kidney disease; PD-CKD, peritoneal dialysis of chronic kidney disease; ESRD, end-stage renal disease; HD-CKD, hemodialysis of chronic kidney disease; Hb, hemoglobin; ΔHb, Hb changes from baseline level; NA, not available; response rate: Hb ≥ 11 g/dl or ΔHb*.

**Table 3:** Roxadustat in clinical trials with CRP.

**Study**	**Year**	**Nation**	**Study cohort**	**Control group**	**Treatment duration**	**Effect of CRP on treatment of anemia with roxadustat**	**Effect of CRP on treatment of anemia with epoetin alfa**
Chen N (53)	2019	China	305 UD patients	Epoetin alfa	26 weeks	(1) Hb level: A vs. B = 11.3 ± 1.0 g/dl vs. 11.2 ± 0.9 g/dl (2) Among A and B, roxadustat raised the level of Hb as much	(1) Hb level: A vs. B = 11.0 ± 0.8 g/dl vs. 10.7 ± 0.9 d/dl (2) A of doses was far less than B of doses
Provenzano R (55)	2016	The USA	90 HD patients	Epoetin alfa	19 weeks	(1) Roxadustat dose requirements were independent of baseline CRP levels	(1) Epoetin alfa dose requirements were related to baseline CRP levels (2) Elevated CRP increased epoetin alfa dose requirements
Provenzano R (56)	2016	The USA	145 NDD-CKD patients	NA	24 weeks	(1) ΔHb: A vs. B = 1.82 ± 1.24 g/dl vs. 1.52 ± 1.14 g/dl (2) CRP levels did not influence roxadustat that induced to increase Hb	NA
Besarab A (57)	2016	The USA	48 HD patients and 12 PD patients	NA	12 weeks	(1) Among A and B, roxadustat raised the level of Hb as much (2) Roxadustat dose requirements were independent of baseline CRP levels	NA

*This table summarizes the effects of CRP on roxadustat in clinical trials. NDD-CKD, non-dialysis chronic kidney diseases; HD, hemodialysis; PD, peritoneal dialysis; CRP, C-reactive protein; A: patients with baseline CRP < 5 ng/ml; B: patients with baseline CRP > 5 ng/ml. ΔHb: mean maximum change in Hb; Hb, hemoglobin; NA, Not available; UD, undergoing dialysis; vs., versus*.

### Roxadustat in Phase III Clinical Trials

#### Roxadustat Treatment for Anemia in Patients Undergoing Long-Term Dialysis (NTC02652806)

The purpose of this phase III clinical study was to evaluate the efficacy and safety of roxadustat in the treatment of anemia in dialysis patient. Three hundred and five patients with end-stage renal disease undergoing dialysis were randomly assigned to roxadustat group and epoetin alfa group. The results showed that roxadustat increased hemoglobin statistically significant non-inferiority relative to epoetin alfa [difference: 0.2 ± 1.2 g/dl; 95% confidence interval (CI): −0.02 to 0.5], The level of hepcidin in the roxadustat group and the epoetin group decreased by 30.2 ng/ml (95% CI: −64.8 to 13.6), and 2.3 ng/ml (95% CI: −51.6 to 6.2), respectively. In addition, patients with elevated CRP levels had similar CRP mean hemoglobin levels and comparable dose in roxadustat group, however, although the dose of epoetin alfa was increased in epoetin group. In the elevated CRP, the hemoglobin level of roxadustat group was larger than that of epoetin alfa group (0.9 ± 1.0 g/dl vs. 0.3 ± 1.1 g/dl) (53).

#### Roxadustat for Anemia in Patients With Kidney Disease Not Receiving Dialysis (NCT02652819)

The purpose of this phase III clinical study was to evaluate the efficacy and safety of roxadustat. One hundred and fifty four patients who did not receive dialysis were randomized to 2:1 to the roxadustat and placebo groups. The results showed a hemoglobin increase of 1.9 ± 1.2 g/dl in roxadustat group and 0.4 ± 0.8 g/dl in the placebo group (*P* < 0.001). The decrease in hepcidin in the roxadustat group was 56.14 ± 63.40 ng/ml and 15.10 ± 48.06 ng/ml, respectively (54).

### Roxadustat Can Reduce Inflammation-Induced Hepcidin

Inflammation interferes with the body's iron homeostasis through hepcidin. Therefore, reducing hepcidin is the key to treating inflammatory anemia (58). Hepcidin antibodies now a new strategy for treating inflammatory anemia (59–61). However, it is well-known that under low oxygen conditions, serum hepcidin in the human body can effectively reduce (62, 63). At the beginning, people thought that it was hypoxia to stabilize HIF which directly inhibited hepatocyte synthesis of hepcidin at the gene level. However, in a hypoxic treatment of HepG2 cells, knockdown of HIF-1α or HIF-2α did not reverse the decline of Hepcidin mRNA, which was not a direct target of HIF-1α or HIF-2α (64, 65).

In experiments conducted by Peyssonnaux et al. mice showed elevated levels of serum inflammatory cytokines IL-6 and IL-1 due to hepatic inflammation and steatosis, whereas decreased levels of hepcidin and increased expression of FNP in mice with VHL gene deletion. Experiments have shown that inhibition of VHL or PHD activity can be used to treat chronic inflammatory anemia (66). Therefore, increased hepcidin caused by inflammation is reduced by inhibiting the activity of VHL and PHD.

Recently, studies have found that hypoxia-lowering hepcidin appeared to be related to platelet derived growth factor (PDGF-B) in bone marrow (27, 67). When PDGF-B or its receptor was inactivated, inhibition of hepcidin was relieved (67, 68), and PDGF-B is a target gene of HIF-1α (69). Or HIF may be an expression of hepcidin controlled by an indirect way (70). Chaston et al. found that hypoxia inhibited the expression of hepcidin by BMP-SMAD pathway (71), but there was no clear explanation for how hypoxia inhibits hepcidin expression.

Roxadustat is a PHI that works under conditions that simulate hypoxia. The study found hepcidin levels was significantly greater in receiving roxadustat than in receiving placebo for CKD of patients (37.5 vs. 4.8 ng/mL, respectively, *P* < 0.0001). Hepcidin level was a dose-dependent decrease fir roxadustat (50). Many clinical studies have shown that roxadustat could significantly reduce hepcidin levels in patients with CKD (50, 51, 53–57). Even though plasma CRP was above the upper limit of normal, roxadustat still reduced plasma hepcidin.

### Roxadustat Is Beneficial to the Intestinal Absorption of Dietary Iron

**Figure 2:**
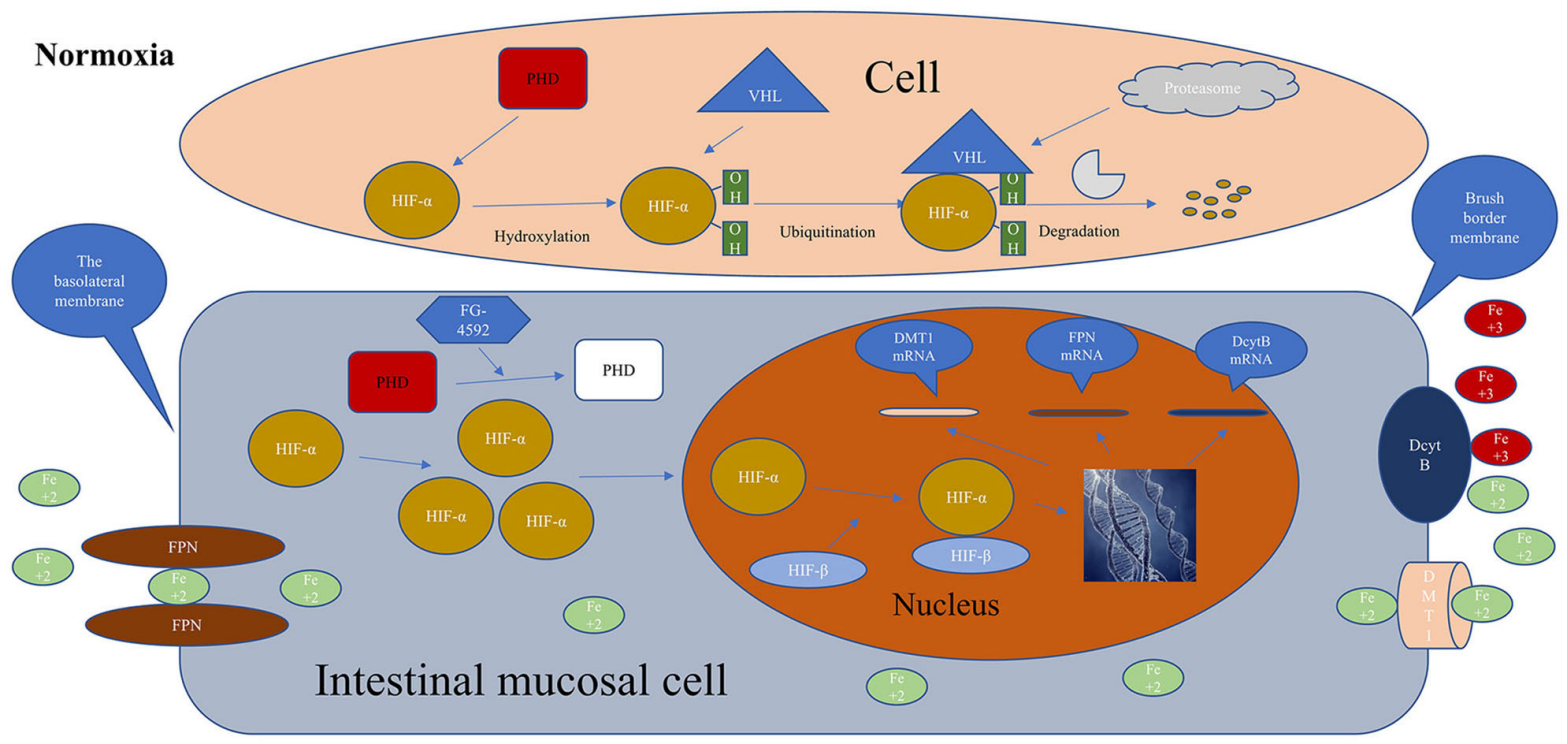
Metabolisms of HIF in the cell without roxadustat, Iron is normally absorbed in the intestines and roxadustat promotes iron absorption. HIF-α, hypoxia-inducible factor α; HIF-β, hypoxia-inducible factor β; PHD, prolyl hypoxia domain protein; FG-4592, roxadustat; FPN, ferroportin; DcytB, duodenal cytochrome B; DMT1, divalent metal transporter 1; VHL, von Hipple-Lindau tumor suppressor; red, activation; white, inactivation; Normoxia, normal oxygen level in the body.

Roxadustat can reduce plasma hepcidin to restore the intestinal absorption of dietary iron. In addition, it has been found that hypoxia could enhance the absorption of dietary iron by the intestines (72). It has been shown that stable HIF-2 could promote the expression of DcytB, DMT1, and FPN at the gene level (73–75), while HIF-1 can promote transferrin expression. In the second phase of the Roxadustat trial conducted by Besarab et al. dialysis patients can significantly increase hemoglobin levels regardless of iron supplementation or iron supplementation. Elevated hemoglobin levels were almost the same for oral iron and intravenous iron, and was not influenced by CRP (57). Therefore, roxadustat can stabilize HIF by inhibiting PHD to promote the expression of DcytB, DMT1, and FPN in mucosal cells, accelerate the absorption of intestinal iron, and promote the release of iron from macrophages of FPN, which is beneficial to supplement plasma iron to meet the demand for iron in erythropoiesis. Figure 2 showed that roxadustat can promote intestinal iron absorption.

### The Effect of Roxadustat on Inflammation

Inflammation and inflammatory cytokines affected erythrocyte, and were also responsible for EPO resistance. It has long been known that early ischemic preconditioning of organs that were ischemia-reperfusion would reduce the damage to organs, which seemed to be related to the hypoxic protection mechanism of the body. In a trial conducted by Cheng et al. early intermittent chronic hypoxic preconditioning of skeletal muscle in rats with ischemia-reperfusion resulted in decreased signs of inflammation such as tumor necrosis factor and macrophage (76), and the hypoxic protective mechanism appeared to have Inhibition of inflammation reduces the effects of ischemia-reperfusion injury (77).

**Figure 3:**
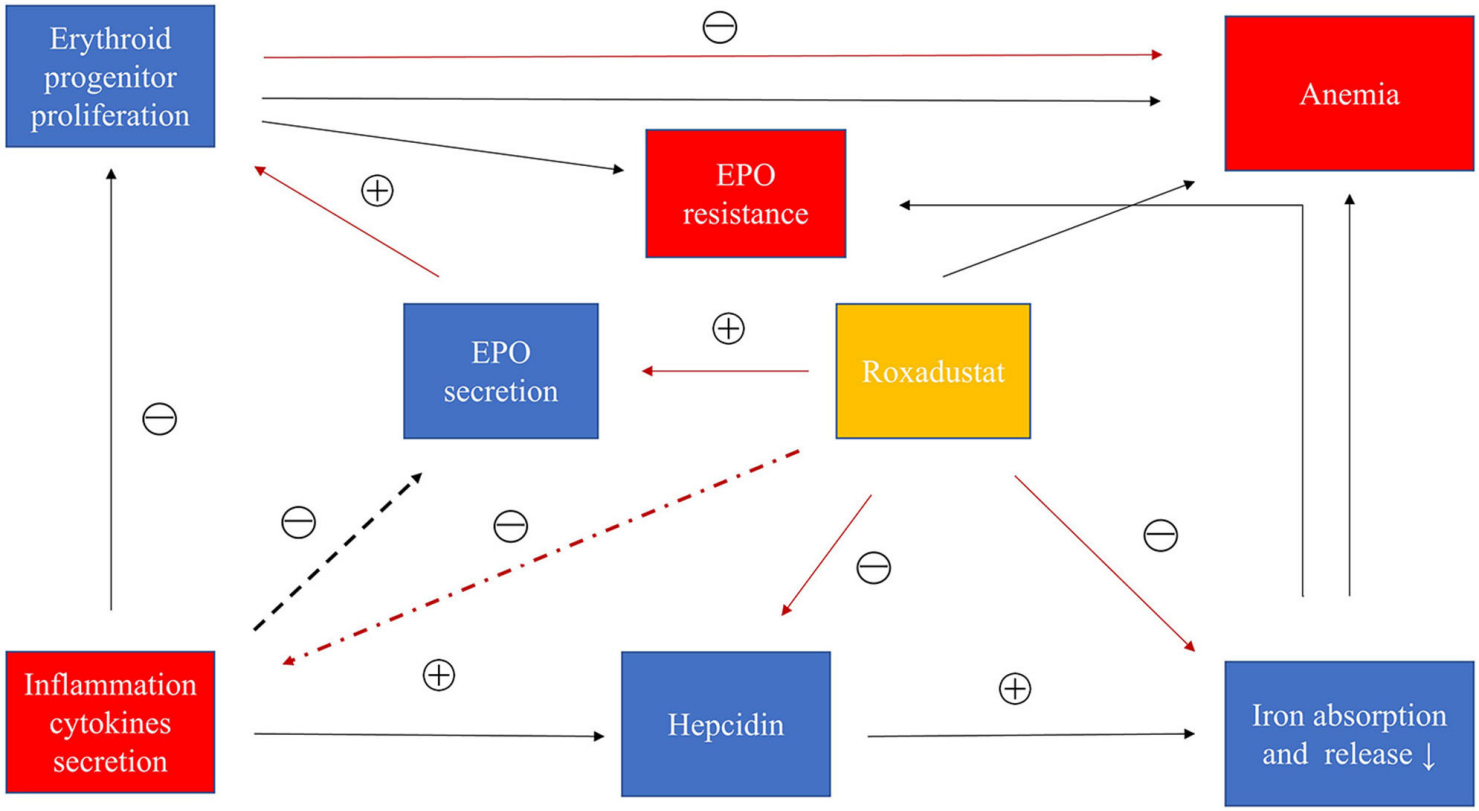
Roxadustat treats inflammatory anemia in CKD. Inflammatory cytokines promote the production of hepcidin to inhibit iron absorption and iron transport and iron release. Inflammatory factors can also interfere with bone marrow hematopoiesis. In addition, inflammatory factors may inhibit the production of EPO. In summary, inflammation causes anemia and EPO resistance through these causes. Roxadustat can inhibit the increase of hepcidin caused by inflammation and promote the production of EPO, and may also inhibit the secretion of inflammatory cytokines. Roxadustat treats inflammatory anemia by these factors. Black: inflammation path; red: roxadustat path.

HIF can promote adenosine gene expression and assist adenosine to exert anti-inflammatory effects (78–80). Many studies have confirmed that HIF had anti-inflammatory and promoted the function of inflammation regression (78, 81, 82). Kobayashi et al. demonstrated that myeloid-specific HIF reduces renal inflammation associated with chronic kidney injury, and that overall loss of HIF or myeloid-specific inactivation promotes inflammation, and Long-term hypoxia in an unimpaired kidney inhibits the expression of multiple inflammatory molecules (83). In addition, it has been found that T cells or dendritic cells lacking HIF-1 cause aggravation of intestinal inflammation (84), especially in inflammatory bowel diseases, and the treatment of inflammatory bowel disease with PHIs has become a new approach (85). In addition, in the kidney injury induced by cisplatin alone, the inflammatory cytokines such as TNF-α, IL-1β, IL-6 were significantly increased, but after treatment with roxadustat and cisplatin, these inflammatory factors were much reduced. The trial confirmed that roxadustat had a potential anti-inflammatory effect and was associated with HIF (86). In the roxadustat phase 3 trial in China, the dose of roxadustat decreased slightly in the CRP above the upper limit group as the treatment time decreased. However, epoetin alpha increased the dose over time, and it was difficult for Hb to reach the target value. From a molecular perspective, roxadustat can stabilize HIF against inflammation and inhibit the production of inflammatory cytokines, but more clinical studies are needed to confirm this possibility. Figure 3 showed the mechanisms of roxadustat in the treatment of inflammatory anemia.

## Safety of Roxadustat in the Treatment of Anemia

A case of association between roxadustat and pulmonary hypertension was reported in Poland. The investigators believed that roxadustat stabilized HIF-2α and affected the production of pulmonary hypertension induced by vascular remodeling. Although the association between roxadustat and pulmonary artery was not clear (87). PHI inhibits PHD from a molecular mechanism, then stabilizes HIF-2α to up-regulate Notch3 and transform growth factor β, and promote the conversion of pericytes into myofibroblasts or vascular smooth muscle cells. This is related to the formation of pulmonary hypertension. Extensive research evidence suggested that pulmonary hypertension was associated with poor long-term viability and increased mortality in patients with advanced kidney disease (88, 89). Therefore, the association between roxadustat and pulmonary hypertension needs further research.

Fortunately, the Phase 2 and Phase 3 clinical trials were currently announced, and roxadustat was well-tolerated, there were no deaths in roxadustat-treated patients. Of course, drug-related adverse events mainly including infection (upper respiratory tract infection, urinary tract infection), hyperkalemia, metabolic acidosis, abnormal liver function, Gastrointestinal disorders (such as diarrhea and vomiting, etc.), Gastrointestinal disorders (Such as diarrhea and vomiting, etc.), the serious adverse events in roxadustat-related subjects included vascular access complications, however, in general, fewer drug-related adverse events occurred (50–57).

## Conclusions

All the above studies show that roxadustat can up-regulate erythropoietin, reduce hepcidin, and promote intestinal absorption of iron by stabilizing HIF. Therefore, it can correct iron metabolism disorders due to inflammation in the body. It may be a new choice for the treatment of chronic kidney disease inflammatory anemia. Of course, roxadustat anti-inflammatory and inhibitory inflammatory cytokine also require more clinical studies to confirm.

## Author Contributions

ZY: conceptualization, software, writing–original draft, investigation, data curation and visualization, and formal analysis. GX: supervision, project administration, and writing–review and editing. All authors contributed to the article and approved the submitted version.

## Conflict of Interest

The authors declare that the research was conducted in the absence of any commercial or financial relationships that could be construed as a potential conflict of interest.

## Abbreviations

EPO: erythropoietin
ESAs: erythropoiesis-stimulating agents
CRP: C-reactive protein
hs-CRP: high sensitivity CRP
CKD: chronic kidney disease
DMT1: divalent metal transporter 1
FPN: ferroportin
DcytB: duodenal cytochrome B
HIF: hypoxia-inducible factor
VHL: von Hipple-Lindau tumor suppressor
PHD: prolyl hydroxylase domain protein
PDGF-B: platelet-derived growth factor B
IL-6: interleukin 6
BMP: bone morphogenetic protein
Hb: hemoglobin
HD: hemodialysis
TNF: tumor necrosis factor
CI: confidence interval.
